# Evaluating the effectiveness of rosuvastatin in preventing the progression of diastolic dysfunction in aortic stenosis: A substudy of the aortic stenosis progression observation measuring effects of rosuvastatin (ASTRONOMER) study

**DOI:** 10.1186/1476-7120-9-5

**Published:** 2011-02-07

**Authors:** Davinder S Jassal, Kapil M Bhagirath, Erin Karlstedt, Matthew Zeglinski, Jean G Dumesnil, Koon K Teo, James W Tam, Kwan L Chan

**Affiliations:** 1 Cardiology Division, Department of Internal Medicine, St. Boniface General Hospital, Winnipeg, Manitoba, Canada; 2 Institute of Cardiovascular Sciences, Cardiology Division, Department of Cardiac Sciences, St. Boniface General Hospital, Winnipeg, Manitoba, Canada; 3 Department of Radiology, St. Boniface General Hospital, Winnipeg, Manitoba, Canada; 4 Hopital Laval, Sainte-Foy, Quebec, Canada; 5 McMaster University, Hamilton, Ontario, Canada; 6 University of Ottawa Heart Institute, Ontario, Canada

## Abstract

**Background:**

Tissue Doppler imaging (TDI) is a noninvasive echocardiographic method for the diagnosis of diastolic dysfunction in patients with varying degrees of aortic stenosis (AS). Little is known however, on the utility of TDI in the serial assessment of diastolic abnormalities in AS.

**Objective:**

The aim of the current proposal was to examine whether treatment with rosuvastatin was successful in improving diastolic abnormalities in patients enrolled in the Aortic Stenosis Progression Observation Measuring Effects of Rosuvastatin (ASTRONOMER) study.

**Methods:**

Conventional Doppler indices including peak early (E) and late (A) transmitral velocities, and E/A ratio were measured from spectral Doppler. Tissue Doppler measurements including early (E') and late (A') velocities of the lateral annulus were determined, and E/E' was calculated.

**Results:**

The study population included 168 patients (56 ± 13 years), whose AS severity was categorized based on peak velocity at baseline (Group I: 2.5-3.0 m/s; Group II: 3.1-3.5 m/s; Group III: 3.6-4.0 m/s). Baseline and follow-up hemodynamics, LV dimensions and diastolic functional parameters were evaluated in all three groups. There was increased diastolic dysfunction from baseline to follow-up in each of the placebo and rosuvastatin groups. In patients with increasing severity of AS in Groups I and II, the lateral E' was lower and the E/E' (as an estimate of increased left ventricular end-diastolic pressure) was higher at baseline (p < 0.05). However, treatment with rosuvastatin did not affect the progression of diastolic dysfunction from baseline to 3.5 year follow-up between patients in any of the three predefined groups.

**Conclusion:**

In patients with mild to moderate asymptomatic AS, rosuvastatin did not attenuate the progression of diastolic dysfunction.

## Background

Aortic stenosis (AS) is the most common valvular heart disease in adult subjects in North America [1,2]. It causes fixed left ventricular (LV) outflow tract obstruction, leading to concentric left ventricular hypertrophy (LVH) and diastolic dysfunction [2,3]. Diastolic dysfunction is characterized by an increased resistance to filling due to increased LV mass, resulting in increased LV end diastolic pressures [2,4]. The inability of the myocardium to relax compromises passive filling of the LV, leading to angina, dyspnea, and pulmonary congestion [2,4].

Tissue Doppler imaging (TDI) is recognized as a noninvasive tool to determine abnormal LV relaxation, and has been shown to be effective in analyzing left ventricular diastolic function in relation to mitral annulus velocity [5-7]. In patients with varying degrees of AS severity, TDI is effective in evaluating diastolic dysfunction [8-12]. TDI has been shown to be effective in evaluating subtle changes in diastolic properties of the heart, even in the absence of LVH [8]. Little is known however, on the utility of TDI in the serial assessment of diastolic abnormalities in patients with AS.

The aim of the current study was to examine whether treatment with rosuvastatin was successful in improving diastolic abnormalities in patients with mild to moderate AS.

## Methods

### Patient Population

Between 2002-2005, 272 patients from 23 Canadian centers with mild to moderate AS were recruited for the Aortic Stenosis Progression Observation Measuring Effects of Rosuvastatin study (ASTRONOMER), a multicenter randomized trial to evaluate the effects of rosuvastatin on the progression of AS [13]. Patients between the ages of 18 and 82 years with mild to moderate AS, defined by a peak Doppler aortic valve velocity of 2.5 to 4.0 m/s, were included. Subjects were divided into three predefined categories of AS severity (peak velocity 2.5-3.0 m/s; 3.1-3.5 m/s; and 3.6-4.0 m/s). The baseline low-density lipoprotein cholesterol complex and triglyceride levels needed to be within target level for all risk categories according to the Canadian Guidelines for patients to be included in the trial [14]. Patients were excluded if they had a very high risk of coronary artery disease (CAD) with an estimated 10 year risk exceeding 30% risk, preexisting diabetes, known CAD, congestive heart failure with New York Heart Association class III or IV symptoms, uncontrolled hypertension (defined as diastolic blood pressure >100 mm Hg or systolic blood pressure > 200 mm Hg), atrial fibrillation or bradyarrhythmias, greater than moderate aortic regurgitation, significant concomitant mitral valve disease, or significant renal impairment (serum creatinine > 200 umol/L) [13]. After the baseline assessment and randomization, the patients were followed up every 3 months to assess for adverse side effects and to ensure compliance. For patients undergoing aortic valve replacement, the measurements on the last echocardiograms before surgery were used in the analysis [13].

For the purpose of the current subanalysis of the ASTRONOMER study, only those individuals with complete and analyzable diastolic echocardiographic parameters at the culmination of the study were included. The final cohort included 168 patients in whom diastolic echocardiographic parameters were available both at baseline and at study end. The study protocol was approved by the institutional review boards at the various participating centers.

### Echocardiography

Parasternal and apical views were obtained using standard echocardiographic systems and multifrequency transducers with tissue Doppler capability. Standard two-dimensional images, M-mode, spectral and color Doppler, and TDI were performed. LV interventricular septal thickness, posterior wall thickness, and LV ejection fraction were determined from two-dimensional images according to established criteria [15]. LV mass was calculated using the cube formula and indexed to body surface area [15]. Heart rate, SBP and DBP were recorded at the time of echo.

Transmitral LV filling velocities at the tips of the mitral valve leaflets were obtained from the apical four-chamber view using pulsed-wave Doppler echocardiography. The transmitral LV filling signal was traced manually, and the following variables were obtained: peak early (E) and late (A) transmitral velocities, E/A ratio, and E-wave deceleration time. Tissue Doppler-derived indices were recorded at the lateral mitral annulus. These indices included systolic velocities (S'), early diastolic velocities (E'), and late diastolic velocities (A'). Finally, the dimensionless index of E/E' was calculated.

### Statistics

The data is summarized as mean ± standard deviation or number (%). Within each arm, placebo and rosuvastatin respectively, baseline and follow-up values were compared using two-sample t-test. To compare changes in the clinical and echocardiographic variables between the two arms (placebo vs. rosuvastatin), all follow-up values were averaged, subtracted from baseline measurements, and the changes were compared using a two-sample t-test or Wilcoxon rank test depending on the distribution of the change. A *p *value less than 0.05 was considered statistically significant. The Statistical Analysis System 8.01 (SAS Institute, Cary, NC), was used to perform the analysis.

## Results

The overall study population included 168 patients [92 males (55%)] with a mean age of 56 ± 13 years, who fulfilled the echocardiographic criteria. The patients were divided into three predetermined categories of AS severity based on peak velocity at baseline (Group I: 2.5-3.0 m/s; Group II: 3.1-3.5 m/s; Group III: 3.6-4.0 m/s). Baseline and follow-up hemodynamics, LV dimensions and diastolic functional parameters were independently evaluated in all three groups.

Baseline and follow-up data comparing the placebo arm to the treatment arm for Group I (2.5-3.0 m/s) are shown in Table 1. Heart rate, systolic and diastolic blood pressures were similar from baseline to follow-up. The severity of AS increased from a peak gradient of 31 ± 3 mm Hg to 42 ± 11 mm Hg in the placebo arm, and 32 ± 3 mm Hg to 43 ± 12 mm Hg in the rosuvastatin arm (p < 0.05 for each arm, respectively). However, the mean difference in progression of AS severity from baseline and follow-up between both arms did not differ (p = 0.60) (Table 1).

**Table 1:** Clinical and echocardiographic findings of Group I patients (n = 64) in the two treatment groups

Characteristics	Placebo (n = 37)		Rosuvastatin (n = 27)		p value
	Baseline	Follow-up	Baseline	Follow-up	
Age, y	56 ± 13		51 ± 11		0.17
*Hemodynamics*					
HR (bpm)	70 ± 8	70 ± 11	70 ± 8	68 ± 10	0.82
SBP (mm Hg)	130 ± 17	131 ± 13	131 ± 20	132 ± 17	0.76
DBP (mm Hg)	77 ± 10	77 ± 13	76 ± 9	74 ± 11	0.79
					
*Aortic Valve Parameters*					
AVA (cm^2^)	1.9 ± 0.7	1.6 ± 0.7*	1.9 ± 0.6	1.6 ± 0.6†	0.62
AV peak velocity (m/s)	2.8 ± 0.1	3.3 ± 0.7*	2.7 ± 0.2	3.1 ± 0.5†	0.57
AV peak gradient (mm Hg)	31 ± 3	42 ± 11*	32 ± 3	43 ± 12†	0.60
AV mean gradient (mm Hg)	17 ± 2	31 ± 3*	17 ± 2	30 ± 4†	0.58
					
*Left Heart Dimensions*					
IVS (mm)	11 ± 2	11 ± 2	11 ± 2	11 ± 2	0.94
PWT (mm)	10 ± 2	11 ± 2	10 ± 2	10 ± 2	0.76
LA (mm)	37 ± 6	37 ± 6	36 ± 6	37 ± 7	0.78
LVEDD (mm)	49 ± 5	49 ± 7	49 ± 7	49 ± 7	0.81
LVESD (mm)	29 ± 4	29 ± 7	29 ± 6	29 ± 6	0.84
LV mass/BSA (g/m^2)^					
Men	95 ± 24	93 ± 23	94 ± 28	95 ± 23	0.61
Women	85 ± 19	87 ± 25	84 ± 24	86 ± 19	0.69
EF (%)	65 ± 6	67 ± 4	65 ± 6	68 ± 9	0.58
					
*Conventional Diastolic Parameters*					
Mitral E velocity (cm/sec)	84 ± 19	88 ± 23	82 ± 20	89 ± 21	0.59
Mitral A velocity (cm/sec)	74 ± 18	76 ± 18	73 ± 25	75 ± 25	0.64
E/A ratio	1.2 ± 0.4	1.1 ± 0.3	1.1 ± 0.4	1.2 ± 0.5	0.71
					
*Tissue Doppler Imaging*					
Lateral S'	8.4 ± 3.2	8.3 ± 1.8	8.5 ± 2.3	8.4 ± 2.1	0.75
Lateral E'	10.2 ± 4.2	8.0 ± 3.2*	10.5 ± 3.5	8.2 ± 3.1†	0.66
Lateral A'	9.2 ± 3.6	9.4 ± 2.8	9.1 ± 2.4	9.4 ± 3.0	0.56
E/E' (lateral)	8 ± 4	11 ± 3*	8 ± 3	11 ± 4†	0.43

Values are mean ± SD (percentage). y, years; SBP, systolic blood pressure; DBP, diastolic blood pressure; HR, heart rate; bpm, beats per minute; AVA, aortic valve area; AV, aortic valve; IVS, interventricular septum; PWT, posterior wall thickness; LA; left atrium diameter in anterior-posterior dimension; LVEDD, left ventricular end diastolic diameter; LVESD, left ventricular end systolic diameter; LV, left ventricular; BSA, body surface area; EF, ejection fraction; S', systolic annular velocity; E', early diastolic annular velocity; A', late diastolic annular velocity. p < 0.05* was considered significant comparing baseline and follow-up values within the placebo arm. p < 0.05† was considered significant comparing baseline and follow-up values within the rosuvastatin arm. Overall p values are listed for the *difference of baseline and follow-up measurements *comparing placebo vs. rosuvastatin, with p < 0.05‡ considered significant.

**Figure 1:**
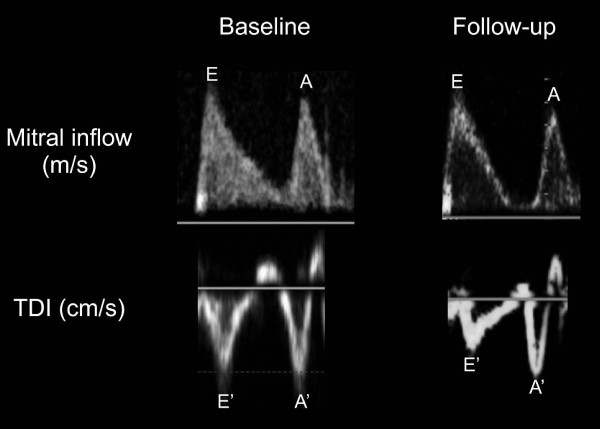
**Diastolic parameters in Group I**. Representative diastolic and TDI parameters at baseline and follow-up in either placebo or rosuvastatin treated groups, demonstrating no change in E/A ratio, but a decrease in the lateral E'.

All 64 patients in group I had conventional and TDI diastolic parameters assessed at baseline and 3.5 year follow-up (Table 1). There was no difference in mean transmitral E-wave, A-wave, nor E/A ratio between both groups. There was a decrease in the lateral E' from 10.2 ± 4.2 cm/s to 8.0 ± 3.2 cm/s in the placebo arm, and 10.5 ± 3.5 cm/s to 8.2 ± 3.1 cm/s in the rosuvastatin arm (p < 0.05 for each arm, respectively), as shown in Figure 1. The lateral E/E' increased from approximately 8 to 11 over time in both placebo and rosuvastatin arms (p < 0.05), respectively. However, there was no difference in progression of diastolic dysfunction from baseline to follow-up at 3.5 years, comparing placebo vs. rosuvastatin (p = 0.43) (Table 1).

In Group II (3.1-3.5 m/s), the peak AS gradient increased from 41 ± 4 mm Hg to 62 ± 23 mm Hg in the placebo arm, and 42 ± 4 mm Hg to 59 ± 14 mm Hg in the rosuvastatin arm (p < 0.05 for each arm, respectively), as shown in Table 2. Although the severity of AS increased to the moderate range within each arm, there was no difference in the degree of progression of AS amongst both groups (p = 0.35). All 63 patients in group II had conventional and TDI diastolic parameters assessed at baseline and follow-up (Table 2). There was no statistical difference in conventional measures of diastolic function between both arms. Although the lateral E/E' increased from 11 to 15 over time within each arm (p < 0.05), respectively, there was no difference in progression from baseline to follow-up at 3.5 years comparing placebo vs. rosuvastatin in Group II (p = 0.62) (Table 2).

**Table 2:** Clinical and echocardiographic findings of Group II patients (n = 63) in the two treatment groups

Characteristics	Placebo (n = 27)		Rosuvastatin (n = 36)		p value
	Baseline	Followup	Baseline	Followup	
Age, y	56 ± 12		59 ± 14		0.38
*Hemodynamics*					
HR (bpm)	68 ± 8	69 ± 12	72 ± 9	71 ± 10	0.54
SBP (mm Hg)	130 ± 17	128 ± 11	130 ± 14	130 ± 15	0.81
DBP (mm Hg)	77 ± 11	74 ± 11	77 ± 11	75 ± 9	0.41
					
*Aortic Valve Parameters*					
AVA (cm^2^)	1.5 ± 0.5	1.1 ± 0.3*	1.5 ± 0.4	1.1 ± 0.4†	0.58
AV peak velocity (m/s)	3.2 ± 0.1	3.9 ± 0.7*	3.2 ± 0.1	3.8 ± 0.5†	0.64
AV peak gradient (mm Hg)	41 ± 4	62 ± 23*	42 ± 4	59 ± 14†	0.35
AV mean gradient (mm Hg)	22 ± 4	35 ± 13*	23 ± 3	34 ± 9†	0.63
					
*Left Heart Dimensions*					
IVS (mm)	11 ± 2	11 ± 2	11 ± 2	11 ± 2	0.95
PWT (mm)	10 ± 1	11 ± 2	11 ± 1	11 ± 2	0.56
LA (mm)	36 ± 6	37 ± 7	39 ± 7	39 ± 7	0.42
LVEDD (mm)	48 ± 6	47 ± 5	48 ± 6	47 ± 6	0.92
LVESD (mm)	29 ± 5	27 ± 5	28 ± 5	27 ± 6	0.36
LV mass/BSA (g/m^2)^					
Men	93 ± 22	94 ± 20	93 ± 24	96 ± 17	0.51
Women	82 ± 17	84 ± 19	81 ± 14	83 ± 19	0.72
EF (%)	66 ± 5	66 ± 6	68 ± 6	68 ± 7	0.89
					
*Conventional Diastolic Parameters*					
Mitral E velocity (cm/sec)	80 ± 15	81 ± 22	81 ± 22	80 ± 27	0.64
Mitral A velocity (cm/sec)	80 ± 27	78 ± 22	80 ± 27	79 ± 28	0.82
E/A ratio	1.0 ± 0.3	1.1 ± 0.2	1.0 ± 0.3	1.0 ± 0.3	0.65
					
*Tissue Doppler Imaging*					
Lateral S'	8.1 ± 1.9	8.1 ± 1.6	8.2 ± 1.8	8.3 ± 2.2	0.72
Lateral E'	7.0 ± 1.2	6.0 ± 2.1*	7.1 ± 3.2	6.1 ± 2.6†	0.66
Lateral A'	10.1 ± 3.1	9.4 ± 3.5	10.6 ± 3.6	10.3 ± 2.3	0.55
E/E' (lateral)	11 ± 4	15 ± 3*	11 ± 3	15 ± 4†	0.62

Values are mean ± SD (percentage). y, years; SBP, systolic blood pressure; DBP, diastolic blood pressure; HR, heart rate; bpm, beats per minute; AVA, aortic valve area; AV, aortic valve; IVS, interventricular septum; PWT, posterior wall thickness; LA; left atrium diameter in anterior-posterior dimension; LVEDD, left ventricular end diastolic diameter; LVESD, left ventricular end systolic diameter; LV, left ventricular; BSA, body surface area; EF, ejection fraction; S', systolic annular velocity; E', early diastolic annular velocity; A', late diastolic annular velocity. p < 0.05* was considered significant comparing baseline and follow-up values within the placebo arm. p < 0.05† was considered significant comparing baseline and follow-up values within the rosuvastatin arm. Overall p values are listed for the *difference of baseline and follow-up measurements *comparing placebo vs. rosuvastatin, with p < 0.05‡ considered significant.

In Group III (3.6-4.0 m/s), the peak AS gradient increased from 56 ± 4 mm Hg to 80 ± 19 mm Hg in the placebo arm, and 56 ± 4 mm Hg to 79 ± 16 mm Hg in the rosuvastatin arm (p < 0.05 for each arm, respectively). There was no difference in progression of AS from moderate to severe amongst both groups (p = 0.85), as shown in Table 3. Of the 41 patients in group III who had conventional and TDI diastolic parameters assessed at baseline and follow-up, the conventional diastolic parameters were similar. Although the lateral E/E' was elevated at baseline in both groups, there was no significant increase identified over the follow-up of 3.5 years (Table 3).

**Table 3:** Clinical and echocardiographic findings of Group III patients (n = 41) in the two treatment groups

Characteristics	Placebo (n = 21)		Rosuvastatin (n = 20)		p value
	Baseline	Followup	Baseline	Followup	
Age, y	58 ± 11		53 ± 16		0.22
*Hemodynamics*					
HR (bpm)	74 ± 11	72 ± 12	71 ± 10	70 ± 7	0.38
SBP (mm Hg)	129 ± 16	127 ± 14	133 ± 15	130 ± 14	0.54
DBP (mm Hg)	80 ± 9	77 ± 10	78 ± 10	76 ± 7	0.62
					
*Aortic Valve Parameters*					
AVA (cm^2^)	1.2 ± 0.4	1.1 ± 0.6	1.3 ± 0.5	1.1 ± 0.5	0.67
AV peak velocity (m/s)	3.7 ± 0.2	4.4 ± 0.5*	3.7 ± 0.1	4.4 ± 0.5†	0.88
AV peak gradient (mm Hg)	56 ± 4	80 ± 19*	56 ± 4	79 ± 16†	0.85
AV mean gradient (mm Hg)	32 ± 5	47 ± 14*	32 ± 4	46 ± 12†	0.76
					
*Left Heart Dimensions*					
IVS (mm)	12 ± 2	12 ± 2	12 ± 2	12 ± 3	0.89
PWT (mm)	11 ± 2	12 ± 2	11 ± 1	11 ± 2	0.58
LA (mm)	36 ± 6	37 ± 7	37 ± 6	39 ± 7	0.58
LVEDD (mm)	48 ± 4	47 ± 5	50 ± 5	49 ± 6	0.86
LVESD (mm)	28 ± 5	27 ± 5	30 ± 5	30 ± 6	0.56
LV mass/BSA (g/m^2)^					
Men	94 ± 19	93 ± 17	94 ± 20	95 ± 11	0.54
Women	81 ± 12	82 ± 15	82 ± 11	81 ± 17	0.68
EF (%)	67 ± 7	67 ± 7	68 ± 8	69 ± 8	0.54
					
*Conventional Diastolic Parameters*					
Mitral E velocity (cm/sec)	83 ± 17	83 ± 26	84 ± 26	83 ± 27	0.44
Mitral A velocity (cm/sec)	80 ± 27	80 ± 29	80 ± 27	79 ± 41	0.77
E/A ratio	1.0 ± 0.2	1.0 ± 0.2	1.0 ± 0.2	1.0 ± 0.3	0.75
					
*Tissue Doppler Imaging*					
Lateral S'	8.1 ± 2.7	8.1 ± 1.7	8.3 ± 1.7	8.2 ± 1.6	0.62
Lateral E'	6.0 ± 1.2	6.1 ± 2.1	6.1 ± 2.4	6.1 ± 1.3	0.74
Lateral A'	10.1 ± 3.1	9.4 ± 3.5	10.6 ± 3.6	10.3 ± 2.3	0.55
E/E' (lateral)	14 ± 3	15 ± 2	14 ± 5	15 ± 4	0.77

Values are mean ± SD (percentage). y, years; SBP, systolic blood pressure; DBP, diastolic blood pressure; HR, heart rate; bpm, beats per minute; AVA, aortic valve area; AV, aortic valve; IVS, interventricular septum; PWT, posterior wall thickness; LA; left atrium diameter in anterior-posterior dimension; LVEDD, left ventricular end diastolic diameter; LVESD, left ventricular end systolic diameter; LV, left ventricular; BSA, body surface area; EF, ejection fraction; S', systolic annular velocity; E', early diastolic annular velocity; A', late diastolic annular velocity. p < 0.05* was considered significant comparing baseline and follow-up values within the placebo arm. p < 0.05† was considered significant comparing baseline and follow-up values within the rosuvastatin arm. Overall p values are listed for the *difference of baseline and follow-up measurements *comparing placebo vs. rosuvastatin, with p < 0.05‡ considered significant.

## Discussion

In this observational sub-study from the ASTRONOMER trial, we sought to determine whether statin therapy improved diastolic dysfunction in patients with mild to moderate AS after a median follow-up of 3.5 years. There was increased diastolic dysfunction from baseline to follow-up in each of the placebo and rosuvastatin arms. In patients with increasing severity of AS in groups I and II, the lateral E' decreased and the E/E' increased over time within each arm, despite no change in LV mass nor LVEF (p < 0.05). However, there was no difference in progression of diastolic dysfunction from baseline to follow-up between patients in all three predefined groups with AS who received placebo versus rosuvastatin. To the best of our knowledge, this is the first clinical investigation that specifically examined the effects of statins on the progression of diastolic dysfunction in patients with AS.

From a pathophysiologic perspective, the obstruction imposed on the LV from the stenotic AV produces systolic wall stress, which in turn leads to concentric LVH [2-4]. Although the increased LV mass may help in maintaining overall systolic function, an impairment in diastolic function arises [2-4]. The spectrum of diastolic abnormalities include increased myocardial stiffness, reduced LV compliance, and elevated left atrial and LV end-diastolic pressures [2-4]. These diastolic abnormalities eventually lead to the clinical manifestations of angina, dyspnea, and pulmonary congestion [2-4].

It has been suggested that statins may have beneficial effects on diastolic parameters, predominantly by attenuating the degree of LVH and cardiac fibrosis in murine models of hypertension [16,17]. *Indolfi et al. *examined the effect of simvastatin on chronic pressure-overloaded left ventricles in Wistar rats [16]. The administration of simvastatin significantly reduced renin angiotensin system (RAS) membrane targeting, RAS in vivo activation, and *ERK2 *phosphorylation, which prevented the development of LVH in this murine model of chronic pressure overload. Similarily, *Luo et al. *demonstrated that simvastatin reduced LVH due to inhibition of local cardiac angiotensin converting enzyme (ACE) activity [17].

A number of clinical studies have evaluated diastolic dysfunction in patients with varying degrees of AS severity [8-12]. We previously demonstrated that in patients with mild to moderate AS (AVA 1.2-1.7 cm^2^), measures of diastolic function were abnormal and related to increasing severity of AS [8]. Specifically, the lateral E' was lower and the E/E' was higher with greater severity of AS [8]. Diastolic abnormalities in a subset of patients with asymptomatic moderate AS were recently examined in the Simvastatin and Ezetimibe in Aortic Stenosis (SEAS) baseline population [9]. The severity of AS in this patient population was defined as an aortic valve area of 1.2 ± 0.4 cm^2 ^and a mean transaortic peak velocity of 3.2 ± 0.5 cm/s [9]. The LV diastolic function was impaired as evident from augment LV filling pressures (measured by septal E/E' and E/Vp) and impaired LV relaxation (measured by reduced septal E') [9]. *Lancelotti et al. *evaluated patients with severe AS (AVA 0.8-0.2 cm^2^; mean gradient 45 mm Hg), in whom an E/E' ratio >14 identified a subset of patients at greater risk of future events [11]. Finally, *Bruch et al. *demonstrated an impairment in diastolic function using TDI in symptomatic patients with advanced aortic AS and LVH [12]. In this small, single centre study, 36 patients with moderate to severe AS were evaluated with a mean AVA of 0.8 ± 0.4 cm^2 ^and mean transaortic pressure gradient of 57 ± 17 mm Hg [12]. *Bruch et al. *demonstrated that the E/E' ratio allows for a reliable and reproducible estimation of LV filling pressures in patients with advanced AS, as compared to age-matched controls [12].

Despite the lack of clinical studies examining the effect of statins on preventing diastolic dysfunction in AS patients, a study by *Fukuta et al. *suggested that statin therapy may lower mortality in patients with diastolic heart failure due to its pleotropic effects in hypertension and CAD [18]. In this non-randomized and non-blinded study involving 137 patients followed for a mean of 21 months, 34 patients with statin therapy were alive at study termination compared with 26 in the placebo group (p = 0.003) [18]. Statin therapy also led to a trend in decreased cardiovascular hospitalizations (p = 0.08) [18]. The findings of this study were clearly hypothesis-generating.

The results of our study are unique in that it is the first to directly evaluate the effects of statins on preventing the progression of diastolic dysfunction in patients with AS. With increasing severity of AS, the degree of diastolic dysfunction, as reflected by a lower E' and higher E/E' ratio, is in agreement with previous studies described above [8-12]. However, as statin therapy did not prevent the progression of AS in multiple randomized controlled studies including ASTRONOMER, SEAS and SALTIRE, [12,19,20] it is not unexpected that the degree of diastolic dysfunction continued to progress in our patient population. Following open surgical or percutaneous aortic valve replacement, it has been shown that diastolic parameters improve in both short and long term follow-up [10,21,22]. It it also plausible that our study population may have other contributing mechanisms to progressive diastolic dysfunction including underlying asymptomatic CAD that could affect the subsequent outcome.

There were some important limitations in the current study. This was an observational sub-study of the ASTRONOMER trial. The original sample size was intended to answer the primary question of whether statins affect the progression of mild to moderate AS, rather than to evaluate the effects of statins on preventing diastolic dysfunction. Given the subgroup nature of these analyses, the results of the current study need to be interpreted cautiously. Although the ASTRONOMER study included 272 patients in total, the current subanalysis study included only 168 patients in whom diastolic echocardiographic parameters were available both at baseline and at study end. Furthermore, the sample size may have been too small to detect true changes even if they exist. Similar to other studies using TDI, the methodology is angle dependent and can be affected by cardiac translation, rotation or both. Additional measurements of diastolic function including medial TDI, LA volumes, strain, strain rate and Vp were not available. Finally, the baseline patients in the ASTRONOMER trial represent a fairly homogenous, Caucasian population, and therefore, it is difficult to extrapolate these results to other ethnic groups.

## Conclusion

In patients with mild to moderate AS, progression of diastolic abnormalities was observed over the 3.5 year follow-up. Rosuvastatin did not attenuate the progression of diastolic dysfunction in this patient population.

## Competing interests

The authors declare that they have no competing interests.

## Authors' contributions

DJ, KM, EK, MZ, JD, LT, JW and KC contributed to the writing of the manuscript. All authors read and approved the final manuscript.
